# Effect of a visual tracking intervention on attention and behavior of children with Attention Deficit Hyperactivity Disorder

**DOI:** 10.16910/jemr.12.8.6

**Published:** 2020-04-22

**Authors:** Shiva Janmohammadi, Hojjat Allah Haghgoo, Mojgan Farahbod, Paul G. Overton, Ebrahim Pishyareh

**Affiliations:** University of Social Welfare and Rehabilitation Sciences, Tehran, Iran; Tehran University of Medical Sciences, School of Advanced Technology, Iran; Exceptional Children Research Institute, Institute of Education, Organization for Education and Planning, Tehran, Iran; Department of Psychology University of Sheffield, UK

**Keywords:** Eye movement, eye tracking, saccades, cognition, attention, Attention Deficit, Hyper-Activity disorder, response inhibition

## Abstract

Attention deficit hyperactivity disorder is characterized by several cognitive and behavioral problems such as inattention and impulsivity, abnormal control of eye movements and relocation, visual fixation and visuospatial perception. There is a link between core motor functions such as oculomotor function and cognition to the extent that the oculomotor system acts as a mediator between the motor and cognitive functions. Therefore, the effects of eye-tracking intervention were investigated on attention in these children. Thirty - nine boys with ADHD, 6 to 10 years of age were recruited and randomized to receive current occupational therapy (control group), or occupational therapy accompanied with eye-tracking exercises (experimental group). They were evaluated using the Conner's Parent Rating Scale, the Continuous Performance Task-2, and the Test of Visual-Motor Skills-Revised before and after the intervention. Significant improvements in the mean scores of cognitive problems (F=9/22), coping behavior (F=6.03) and hyperactivity (F=9.77) were detected in the posttest between the two groups (p<0.05). Furthermore, in the Continuous Performance Test scores, detectability (F=5.68), omission errors (F=17.89), commission errors (F=19.45), reaction time (F=8.95), variability (F=7.07), and preservation (F=6.33) showed significant differences between control and experimental groups (p<0.01). It appears that eye-tracking interventions designed based on the isolation of neck and eye movement might have an important role in improving cognitive function and coping behaviors in these children. It seems that these exercises could increase eye movement control; improve cognitive function and response inhibition.

## Introduction

Attention deficit hyperactivity disorder (ADHD) is one of the most common psychiatric disorders [1], with a childhood incidence of 2 to 7 percent [2]. ADHD is characterized by having general signs of hyperactivity, impulsivity, and inattention. Researchers have shown that children with ADHD suffer from numerous executive function problems, especially in aspects of attentional performance such as sustained attention, divided attention, and flexibility [3, 4]. The disorder impairs normal functions such as academic success, behavior at school, interaction with parents, siblings, and peers [5]. Children with ADHD demonstrate severe deficits in response inhibition as a core feature of impulsivity [1, 4, 6, 7]. The inability to control responses leads to disturbances in executive function, oculomotor function, and other aspects of motor and cognitive control [8]. In addition to inattention, impulsivity [6], and abnormalities in self-regulation [8], children with ADHD show abnormalities in the control of saccadic eye movements, abnormal eye movements and problems in visual fixation [7, 9], and smooth pursuit movements [10, 11, 12, 13, 14, 15]. Eye-tracking and sustained attention in children with ADHD are impaired with respect to normal children [9]. They suffer from motor problems because of dysfunction in brain areas such as the cerebellum and frontostriatal circuit [16, 17].

Brain imaging studies in patients with ADHD show structural and functional abnormalities in prefrontal and striatal structures [18]. In children with ADHD, brain size and the pattern of neural activity in the parts of the brain that are responsible for response inhibition, eye movements, and cancelling movement commands when gaining control over movements, are different from normal children [7, 19, 20]. This will cause the performance of these functions to be impaired and inadequate [21]. The oculomotor system acts as a mediator between motor and cognitive functions [22, 23]. In children with ADHD, the oculomotor system shows deficits at different brain levels including the basal ganglia [24], thalamus [25], cerebellum [26], brainstem [27], reticular formation [28] and superior colliculus [29]. Children with ADHD have special problems in inhibition and control of eye movements. They tend to eye track for shorter periods and make more errors in the number of saccadic movements, especially when the task takes a considerable time [30, 31]. Children with ADHD are less able to control unwanted saccadic eye movements and one of the most important aspects of the deficit in the control of eye movements is during inhibition of return [7]. The oculomotor deficiencies in children with ADHD result in slower processing speeds, lower levels of automatic skills and more effort in doing tasks [32]. It has also been shown that the type of emotional valance in images (pleasant, unpleasant, neutral) affect eye movements in visual search, and the duration of eye gaze and also eye control [33].

According to the "cognitive-energetic model", there are mutual interactions between disorganized motor behavior, management of goal-directed behavior and correcting errors resulting from behavioral output. On the other hand, based on the sensory integration approach (SI), oculomotor control and visuospatial ability are considered to be a foundation for praxis and planning [34]. In recent years, interventions and strategies to resolve the problems of children with ADHD are amongst the most important research topics. Because the brain structures which control the oculomotor movements are impaired in children with ADHD [10, 13, 35], it was hypothesized that eye movement exercises to promote control might improve cognitive functions in these subjects. Previous researchers have reported that some special visual and attentional exercises based on eye-tracking and visual fixation has reduced the rate of impulsivity and inattentiveness [36]. One study examined the effect of the background on attention. In this study, the child had to look at a moving light and press a button if there was any change in the light, with the experiment conducted in a bright room or a dark room. The results showed that when the task was performed in a dark room, cerebellar involvement in smooth pursuit eye movements was greater. There was a significant decrease in errors in eye movement when performance took place in darkness [12]. In another study that used visual search exercises, there were more non-purposeful saccadic eye movements in ADHD children in comparison with normal ones [37]. Other research indicates that there is a relationship between eye movements and attention [35].

It has been observed that when a stimulus is beyond the accessible space for the individual, there is a need for a great concordance between neck and trunk muscles accompanying eye movements. However, when the stimulus is close to the body, the concordance between the eye movements proves sufficient [38].

In the present study we used an appealing play format to increase the motivation of children with ADHD to completing the exercise. Because of motivational issues, we could not use a more constrained and rigid paradigm in these children. Therefore, eye movement practices and their inhibitory role in behavioural function was investigated in children with ADHD.

## Methods

### Participants

The study was approved by the ethical committee of the Social Welfare and Rehabilitation Sciences University (Code: USWR.AC.IR. 1392.114). Written informed consent was obtained from all participants' caregivers (parents) prior to the study. In total, 53 children aged 6-10 years old were recruited through the "Roshd Occupational Therapy ‎Center” in Tehran, Iran. The subjects were diagnosed with ADHD according to DSM-IV-TR by a child psychiatrist. The child psychiatrist used a clinical interview ‎and also a semi-structured questionnaire ‎based on ‎DSM-IV-TR‎‎, an IQ test, and the K-SADS-PL (Persian version) questionnaire for examining comorbidity. Subjects with a history of other psychiatric or neurological disorders were excluded from the study. These disorders included bipolar disorder, pervasive developmental disorders, cerebral palsy, epilepsy, or mental retardation (IQ below 70 in Wechsler Intelligence Scale-IV), severe visual impairment, medication (except methylphenidate – ‘Ritalin’).

Finally, a total of thirty-nine participants (boys), were recruited and randomized using numbered containers to receive conventional occupational therapy plus visual eye-tracking exercises (20 subjects), referred to as the experimental group, and only conventional occupational therapy (19 subjects), referred as the control group.

The participants were all under the supervision of a child psychiatrist. It ‎was ‎their responsibility to determine the dose of any medication being taken, and no ‎significant ‎changes were reported by the end of the intervention period in ‎the dose ‎of the drugs being taken. One of our inclusion criteria was the use of only ‎one kind of ‎a drug that is Ritalin in our research. All the children who used any ‎other drugs ‎were excluded from the research. Any client with any changes to the ‎dose of the ‎drug or the addition of a new drug was also excluded from the study.‎ Due to changes in medication, inability to follow the program or irregular participation; some of the participants (three subjects in the experimental group, and four subjects from the control group) were excluded.

### Design

During the intervention period, all the subjects received a perceptual-motor programme [39] together with common pediatric massage therapy[40] as a conventional occupational therapy intervention for children with ADHD [34]. However, the subjects in the experimental group additionally participated in the eye-tracking program for 5 weeks, 2 sessions per week, 30 min per session. 

Subjects in both groups were evaluated using the Wechsler Intelligence Scale for Children, the parents' form of Conner's Parent Rating Scale, the Continuous Performance Test (CPT-II) and the Test of Visual-Motor Skills (TVMS) before and after the intervention.

### Materials

#### Wechsler Intelligence Scale for Children

The Wechsler Intelligence Scale for Children (WISC) was developed by David Wechsler in 1942 and revised in 1972. This scale measures the intelligence of children aged 6 years to 16 years, 11 months and 30 days. This scale consists of two main components and 12 subtests. The tests of the verbal skills of children include comprehension, calculation, analogies, vocabulary and number memory. Practical scale tests include completing images, adjusting images, cube design, assembly, encoding (the equivalent numeric codes in adults), and maze.

### Continuous Performance Test

The continuous performance test (CPT) has a long‏ ‏history of use for ‎measuring processes related to sustained attention, vigilance, response ‎inhibition, signal detection, and other aspects of performance. Most of ‎the CPT studies follow the basic paradigm described by Rosvold and Mirsky [41] in which the subject ‎must press a key in response to a rare target, such as the letter “X.” Variations ‎of this paradigm include an “A–X” task in which the target is an “X” ‎preceded by an “A” or other specific letter combinations. Only the target digit of 'X" was used in this study. We used the CPT-II, considered to be a valuable ‎assessment tool that can reveal important information about individual ‎functioning. Omission and commission errors and mean hit reaction time (RT) are the major variables of this test. This test also measures parameters such as preservation, detectability and hit rate error [37].

### Visual-Motor Skills Test-Revised

The Visual -Motor Skills Test (TVMS) was revised by Gardner and Morrison in the United States of America in 1995 and is independent of race, sex, culture and geographical location. This test [42] is used as a means of determining the impairment of eye-hand coordination, and in the diagnosis of children with visual-motor disorders. It can also be used as an outcome measure to document the progress of children in the development of fine visuo-motor skills, for children aged between 3-14 years old. The test is made up of 23 geometric designs and shapes that each occupy one page. The subject is asked to draw and copy the model of these designs. Among Iranian children, the consistency factor for the test is reported as 0.99, test reliability is 0.94, and the validity of the total test is 0.99.

In the study, all assessments were done by examiners blind to the subject's assigned group. Data were analyzed using SPSS software version 20. Levene’s test and t-test were used to examine the consistency of data. The Kolmogorov-Smirnov test was used to evaluate the normality of data. According to the results, it was considered appropriate to use parametric tests (analysis of covariance) to compare the impact of interventions on changes in the two groups. The significance level for all tests was 0.05.

### Procedure

Considering the inclusion criteria and after gathering the consent form, a demographic questionnaire and a Conner's Parent Rating Scale were filled out by the subjects’ caregivers. The children were examined with the Wechsler Intelligence Scale for Children )WISC-IV) and the Test of Visual-Motor Skill (TVMS). In the next stage, CPT-II was undertaken. It should be noted that to avoid any bias during testing, intelligence test assessment, the continuous performance test, and the visual-motor skills test were conducted by a Master of Psychology student at the Institute of Cognitive Sciences Studies (ICSS). After the tests, the subjects were individually treated in a room (3 x 4 meters) in the “Roshd Occupational Therapy Clinic”. The participant was located in the center of the room. The room was quiet, well ventilated, with appropriate temperature and atmospheric controls. Subjects received the eye-tracking intervention for 5 weeks, 2 sessions per week for 30 minutes. Immediately after ending the intervention, the final assessment was completed, including the Conner's Parent Rating Scale completed by caregivers, the TVMSV, as well as the CPT-II.

Practices used in the eye-tracking intervention ‎in this study were based on findings about the inhibitory control of eye movements during oculomotor ‎countermanding [7], stopping performance [43], repeated task-switching‎ [44, 45], inhibition and error detection [46], and executive control of countermanding saccades [47].

Our visual tracking/pursuit paradigm is based on visual fixation (gaze) and ‎head control, and control of the speed of eye movement. We designed our task based on neuronal control systems that keep the ‎eyes on target including saccadic eye movement, smooth pursuit, convergence ‎movements, and the vestibulo-ocular reflex (VOR). The fixation system holds the ‎eye stationary during gaze when the head is not moving. This requires ‎active suppression of eye movements. The gaze system generates eye movements by considering the activity of an oculomotor neuron during a ‎saccade. To move the eye quickly to a new position in the orbit and keep it ‎there, two passive forces need to be overcome: First, the elastic force of the ‎orbit, which tends to restore the eye to a central position in the orbit, and second, a ‎velocity-dependent viscous force that opposes the rapid movement. Thus, the ‎motor signal must include information about the tonic position, which opposes ‎the elastic force, as well as velocity, which overcomes orbital viscosity and moves ‎the eye quickly to a new position. Because the head has much greater inertia ‎than the eyes, a small shift in gaze drives the fovea to its target before the ‎head begins to move. A small gaze shift usually consists of a saccade ‎followed by a small head movement during which the VOR ‎moves the eyes back to the center of the orbit in the new head position. For larger ‎gaze shifts, the eyes and the head move simultaneously in the same direction. Because the VOR ordinarily moves the eyes in the direction ‎opposite that of the head, the reflex must be temporally suppressed for the eyes ‎and head to move simultaneously [48]. Therefore, fundamental components of ‎our exercises focused on the duration of fixation (gaze), the range of eye movement in foveal and ambient visual processing, a Go-No go task involving orientation and direction of eye pursuit and eye-hand coordination.

All of the exercises focused on the following issues:

a) Isolation of neck and eye movement (head stabilization) to promote stability of posture, because visual perception in ADHD is affected by fidgeting and hyperactivity.

b) Promotion of awareness about eye movements and pursuit by the utilization of variation in the orientation and direction components in exercises.

c) Promotion of time perception and patience, because children with ADHD suffer from time aversion and temporal dysregulation that emerges as commission errors in sustained attention tests, for example, the CPT (continuous performance test).

d) Promotion of attention to the environment and working memory to give priority and value to important items and disregard unwanted stimuli to decrease distractions.

A detailed description of these exercises are (all exercises were done while the child was standing in the center of the room, two meters away from a screen board except the exercise titled Distance Drawing, Number 9:

1. Isolated neck and eye movement ‎(tracking and pursuit) ‎ together with vestibular exercises on a tilt board in a standing position: In these tasks, the child holds their head in a fixed position and follows a moving target (laser pointer), reporting what stimuli suddenly appear on a screen and where they were located.

2. Tracking simple and colourful / unicolor ‎/complicated lines in vertical, horizontal or diagonal orientation respectively with a laser pointer: The child holds their head in a fixed position (while standing) and follows lines with a laser pointer based on verbal instructions from the therapist related to stimulus position and speed.

3. Number-based saccadic task with presentation-speed variation (slow, medium, and fast): Numbers randomly appear at the center of a screen and children are asked to name the numbers as soon as they see them.

4. Reporting on images during distraction: Four colored light bulbs are automatically switched on and off every 5 s at 4 corners of a screen. At the same time, pictures are displayed at different orientations on the screen and the child has to name the picture or search in the picture for the requested component. During this activity, the child's head is held in a fixed position by a chin board.

5. Search of the environment by the torch in darkness: The therapist places cards and toys in the room including pasting pictures on the walls and then asks the child to find them in the dark with a torch by a segmental and categorical active search.

6. Shadow play with the torch: the child must coordinate body, head, and eye movements as they cast their shadow or the shadow of toys and instruments on the board, following the therapist's instructions.

7.Fixation practice: The child has a laser pointer in their hand and a headlight attached to their head. The therapist asks the child to hold both lights on a single image or given points and items in a presented picture.

8. Two light configurations: The child's head is held in a fixed position by a chin board. The therapist asks the child to move their eyes in the direction of a light and follow the direction and speed of the light with a torch in a maze (simple, curved and zigzag pathway). The light position is randomized with respect to side.

9. Simple to complex distance drawing by use of a video projector projecting images onto a screen: In this task, a video camera is situated under a table, transferring the image of what a child is drawing on their blackboard to screen. The child is only allowed to look at the screen to complete the task (drawing or completing a picture) on the blackboard while unable to see their own hand.

10. Visual-motor training using the Nintendo Wii Kinect game: Using the Wii fit plus series with a balance board (beginner and intermediate level).

Every session was 30 min long, during which 3-4 types exercise were done. All of the participants did all types of exercise task.

The exercise progressed from simple to complex (difficult). We tried to use an interesting format (play) for the exercise, as play can increase the level of interest the children have and help them complete the tasks o the end. This was the main reason why we could not use a more constrained and rigid paradigm for the intervention in these children.

## Results

Of 53 male subjects recruited as children with ADHD, 39 were included in the research and randomly assigned to the two groups, an experimental group (n=20) and a control group (n=19). The mean age in the control group was 7.37±1.01 and in the experimental group 7.05±0.8. The mean scores of all subjects on the Connors' Parent Rating Scale are presented in Table 1.

**Table 1:** The mean and standard deviation of Attention Deficit Hyperactivity Disorder components in pretest and posttest of Conner's Parent Rating Scale.

Characteristics		Experiment Group		Control Group	
		M	SD	M	SD
Detectability	Pre.T	0.14	0.26	0.22	0.26
	Post.T	0.34	0.21	0.24	0.18
Omission error	Pre.T	35.70	22.85	41.42	22.79
	Post.T	14.45	11.06	35.00	23.03
Commission error	Pre.T	28.60	8.59	25.58	5.33
	Post.T	19.35	5.01	23.68	5.11
Reaction time	Pre.T	600.99	193.43	600.98	224.28
	Post.T	400.22	105.49	660.07	238.56
Variability	Pre.T	40.39	24.32	25.11	23.56
	Post.T	17.88	10.25	22.77	24.24
Preservation	Pre.T	30.50	18.35	17.95	9.96
	Post.T	11.65	8.62	15.95	9.32

Table 2 shows the difference between the mean performance on all subscales of the CPT-II between pre- and post-test, and between the experimental and control groups. As can be seen, in the experimental group the mean average performance posttest is higher than the pretest for all categories. Also in the experimental group, average omission errors, commission errors, reaction times, flexibility and preservation in the post-test were lower than pre-test.

**Table 2:** The mean and standard deviation of Attention Deficit Hyperactivity Disorder components in pretest and posttest of Continuous Performance Test.

Characteristics		Experiment Group	Control Group
		Mean ±SD	Mean ±SD
Cognitive problems	Pretest	16±1.75	15.05±1.81
	Post-test	10.10±2.75	12.48±3.13
Coping behavior	Pretest	10.25±2.95	9.31±3.93
	Post test	5.70±2.08	9.79±6.71
Hyper activity	Pre test	11.65±3.92	12.05±5.01
	Post-test	6.90±3.90	10.84±4.13
Overall	Pretest	37.90±5.72	36.42±6.78
	Post-test	22.70±6.46	33.47±10.67

**Table 3:** Mean and standard deviations of visual-motor skills in pretest and posttest.

	Experimental group		Control group	
	M	SD	M	SD
Pre test	95.26	9.16	95.22	9.33
Post test	114.9	10.25	97.11	9.67

Before using MANCOVA, a parametric test, to comply with its assumptions, and Levene’s and Box’s tests were used. Box’s test at posttest was not significant for any of the variables (p=0.66, F=0.69, Box=4.68), hence demonstrating homogeneity of the variance-covariance matrices. Levene’s test (Table 4) at posttest was non-significant for all variables, hence the equality of variance between groups was respected. So multivariate parametric statistics were deemed to be appropriate. MANCOVA on Attention Deficit Hyperactivity Disorder component scores in both experimental and control groups at posttest, using pretest scores as a covariate, showed significant differences. Wilks Lambda indicates that there are significant differences on at least one of the dependent variables posttest (P<0.01-F=5.82). Also Eta square values show significant differences between the two groups, with the amount of the difference in the posttest based on Wilks lambda of 35%, meaning that 35 % of the variance of the difference between the two groups is due to the interaction of the dependent variables.

**Table 4:** Results of Levene’s test for equality of variances of the two groups in the scores of attention deficit hyperactivity in post-test.

Characteristics	F	Significance level	The second degree of freedom	The first degree of freedom
Cognitive problem	0.002	0.97	37	1
Coping behavior	3.43	0.07	37	1
Hyper activity	0.087	0.77	37	1

As results in Table 5 show, there is a significant difference (p<0.05) between the experimental and control groups in terms of posttest mean scores related to cognitive problems (F=9.22), coping behavior (F=6.03) and hyperactivity (F=9.77). In other words, eye movement intervention skills caused cognitive problems, coping behavior and hyperactivity in children with ADHD to significantly improve at post-test.

**Table 5:** Summary results of the analysis of covariance to the effect on eye movement's intervention to reduce symptom characteristics of ADHD boys.

Characteristics	Source change	SS	df	F	P	Etta square
Cognitive problem	group	66.41	1	9.22 ^ ** ^	0.01	0.21
Coping behavior	group	130.64	1	6.03 ^ * ^	0.05	0.15
Hyper activity	group	111.39	1	9.77 ^ ** ^	0.01	0.22

P<0.001^*** ^<0.01P^**^0.05>P^*^

Again, before using MANCOVA to analyse the CPT-II data, Levene’s and Box’s test were used. Box’s test at posttest (P-29, F-1.15, Box 30.58) was not significant for any of the variables, supporting the assumption of homogeneity of variance of the matrix/covariance. Likewise, Levene’s test at post-test was non-significant for all variables, hence demonstrating equality between-group variance. The Results of the MANCOVA on the components of CPT-II scores in both experimental and control groups at the posttest showed significant effects.

As the results in Table 6 show, despite controlling for the effect of pre-test, there are significant differences between the two groups (P<0.05) at post-test in the average detectability score (F=5.68), omission errors (F=17.89) and commission errors (F=19.45), reaction time (F=8.95), variability (F=7.07) and preservation F=6.33) control test. In other words, the training component of the visual patterns continuous performance test in ADHD significantly improved performance at post- test.

**Table 6:** Summary results of covariance analysis on the effects of education on improving the visual patterns of continuous performance test components boys with ADHD.

Charectristics	Source change	MS=SS	F	Eta square
Detectability	group	0.162	5.68 ^ * ^	0.15
Omission error	group	1908.4	17.89 ^ *** ^	0.37
Commission error	group	300.81	19.45 ^ *** ^	0.39
Reaction time	group	66.12	8.95 ^ ** ^	0.22
Variability	group	1047.58	7.07 ^ * ^	0.19
Preservation	group	324.32	6.33 ^ * ^	0.17

In all variables DF=1, SS=MS,^***^P<0.001, <0.01P^**^0.05>P^*^

Levene’s test for homogeneity of variance on tests of visual-motor skills at post-test showed the homogeneity of variances of visual-motor skills for the total score. Therefore, again MANCOVA was permitted and the results of pretest was considered as a covariate variable. The results of covariance analysis on the effects of education on improving the visual patterns of visual-motor skills test in subjects showed a significant difference in the results between the two groups (p<0.001). Chi Eta is equal to 0.53, evidencing a large effect size. In other words, teaching visual patterns improve visual motor skills and was significantly effective in boys with ADHD.

## Discussion

Modifying anti-saccadic movements in children with ADHD influences their cognitive ability [49] and reduces symptoms of hyperactivity and response inhibition in these children. This effect is similar to the effects of methylphenidate. According to the present study, using eye movement practices leads to decreasing and inhibition of unwanted saccadic eye movements. It seems that visual tracking interventions modify behavioral symptoms according Conner's Parent Rating Scale. Previous studies suggested that abnormalities in eye movement inhibition leads to impulsivity and hyperactivity in children with ADHD [50]. Considering the relationship between the prefrontal cortex and the oculomotor system and inhibition, it seems that the use of oculomotor exercises may reduce hyperactive behavior because it involves similar brain regions. The role of the frontal cortex in synthesizing and integrating data for significant cognitive processes and behavioral patterns have been demonstrated [51]. Given that children with ADHD have dysfunction in the frontal regions such as the prefrontal cortex, especially the dorsolateral prefrontal cortex, and since the same neural circuits control eye movements, it seems that using visual tracking interventions could lead to the behavioral changes in children with ADHD via an interaction with the prefrontal cortex.

The integration of visual and motor systems is the main factor in academic capabilities such as drawing and writing. Therefore, behavioral researchers try to identify, eliminate, or reduce problems in the field of visual-motor skills in children with specific learning disabilities. Visual-motor skills coordinate and accommodate visual processing skills, fine motor skills, and eye-hand coordination. Visual information processing skills are divided into two groups:

A) Visual-spatial skills

B) Visual-motor skills or visual-motor integration [52]

Since the development of these skills interact with each other, therefore, visual-motor skills should also be considered and also eye fixation, which includes the ability to keep the eyeball focused on objects. The ability to attend to details plays an important role in the development and proper functioning in visual-motor skills. When the object is attractive, we move our eyes reflexively to the object. Dependent on the object, if it evokes eye movements, it eventually leads to a greater response in the parietal cortex. So, it seems that there is a close relationship between eye movements and attention [53].

Stimulation of the frontal cortex results in physiological and behavioral effects on attention [54]. Other researchers have found similar results [55, 56]. These results led us to the hypothesis that attention and eye movement work in a coordinated fashion. According to the results of this study, it appears that visual tracking interventions can be used as a therapeutic method. According to the theories of visual tracking and considerable evidence in the literature, visual-motor skills, and visual tracking skills are fundamental to behavioral functions [52]. It is suggested that the study could be replicated in a larger population with a longer duration of treatment either in an ADHD group or in other psychiatric disorders in children with learning difficulties using more specific visual tracking exercises.

 In conclusion, using a rehabilitation program based on visual-motor skills or eye tracking is a promising therapeutic exercise to improve symptoms in children with ADHD.

If we could continue the programme for a long time, we could probably see the effect of this intervention on other factors such as sustained attention and it may be possible to measure the effect of response inhibition on the generalization of social behaviors in these children. It could have a long-term impact on reading and writing skills if the programme continued for a longer duration.

### Limitations

Our results must be considered in light of several limitations. Our sample sizes were not large and we are reporting results only for boys, rather than for combined groups of both sexes. In addition to the common problems in human research, maintaining the treatment process and maintaining specific conditions based on the inclusion criteria limited the research workflow.

Unfortunately, during the research, we did not have access to an eye tracker. If we had accessed an eye tracker, we could have analyzed eye movement patterns much more precisely. 

### Ethics and Conflict of Interest

The author(s) declare(s) that the contents of the article are in agreement with the ethics described inhttp://biblio.unibe.ch/portale/elibrary/BOP/jemr/ethics.html and that there is no conflict of interest regarding the publication of this paper. 

### Acknowledgments

This research was supported in part by the University of Social Welfare and Rehabilitation Sciences, Tehran, Iran. We acknowledge the children with ADHD, their parents and the therapists in the Roshd Center of Rehabilitation who helped and support us kindly to perform the research.

## References

[b35] AnaCubillo, R. , ( 2012). A review of fronto-striatal and fronto-cortical brain abnormalities in children and adults with Attention Deficit Hyperactivity Disorder (ADHD) and new evidence for dysfunction in adults with ADHD during motivation and attention. cortex 194–215. 21575934 10.1016/j.cortex.2011.04.007

[b21] Armstrong, I. T. , & Munoz, D. P. ( 2003). Inhibitory control of eye movements during oculomotor countermanding in adults with attention-deficit hyperactivity disorder. Experimental Brain Research, 152( 4), 444–452. 10.1007/s00221-003-1569-3 0014-4819 12879174

[b44] Aron, A.R. , T.W. Robbins , and R.A.J.T.i.c.s. Poldrack , Inhibition and the right inferior frontal cortex. 2004 8(4): p. 170-177. 10.1016/j.tics.2004.02.010 15050513

[b36] Asiry, O. , Shen, H. , & Calder, P. ( 2015). Extending attention span of ADHD children through an eye tracker directed adaptive user interface. Proceedings of the ASWEC 2015 24th Australasian Software Engineering Conference.

[b24] Aylward, E. H. , Reiss, A. L. , Reader, M. J. , Singer, H. S. , Brown, J. E. , & Denckla, M. B. ( 1996). Basal ganglia volumes in children with attention-deficit hyperactivity disorder. Journal of Child Neurology, 11( 2), 112–115. 10.1177/088307389601100210 0883-0738 8881987

[b34] Ayres, A.J. , Robbins, J. ( 2005). Sensory integration and the child: Understanding hidden sensory challenges. Western Psychological Services,

[b25] Bailey, T. , & Joyce, A. ( 2015). The role of the thalamus in ADHD symptomatology and treatment. Applied Neuropsychology. Child, 4( 2), 89–96. 10.1080/21622965.2015.1005475 2162-2965 25748775

[b4] Barkley, R. A. ( 1997). Behavioral inhibition, sustained attention, and executive functions: Constructing a unifying theory of ADHD. Psychological Bulletin, 121( 1), 65– 94. 10.1037/0033-2909.121.1.65 0033-2909 9000892

[b41] Beck, L. H. , Bransome, E. D., Jr., Mirsky, A. F. , Rosvold, H. E. , & Sarason, I. ( 1956). A continuous performance test of brain damage. Journal of Consulting Psychology, 20( 5), 343–350. 10.1037/h0043220 0095-8891 13367264

[b55] Bushnell, M.C. , M.E. Goldberg , and D.L.J.j.o.N. Robinson ,1981 Behavioral enhancement of visual responses in monkey cerebral cortex. I. Modulation in posterior parietal cortex related to selective visual attention., 46(4),755–772. 10.1152/jn.1981.46.4.755 7288463

[b12] Bylsma, F. W. , & Pivik, R. T. (1989).The effects of background illumination and stimulant medication on smooth pursuit eye movements of hyperactive children. Journal of Abnormal Child Psychology,17(1),73–90. 10.1007/BF00910771 0091-0627 2926024

[b14] Cairney, S. , Maruff, P. , Vance, A. , Barnett, R. , Luk, E. , & Currie,J. (2001).Contextual abnormalities of saccadic inhibition in children with attention deficit hyperactivity disorder. Experimental Brain Research,141(4),507–518. 10.1007/s002210100890 0014-4819 11810144

[b10] Castellanos,F. X. , Marvasti,F. F. , Ducharme,J. L. , Walter,J. M. , Israel,M. E. , Krain,A. , Pavlovsky,C. , & Hommer,D. W. (2000).Executive function oculomotor tasks in girls with ADHD. Journal of the American Academy of Child and Adolescent Psychiatry,39(5),644–650. 10.1097/00004583-200005000-00019 0890-8567 10802983

[b28] Chelune,G. J. , Ferguson,W. , Koon,R. , & Dickey,T. O. (1986).Frontal lobe disinhibition in attention deficit disorder. Child Psychiatry and Human Development,16(4),221–234. 10.1007/BF00706479 0009-398X 3743175

[b42] Coallier,M. , et al.,Visual-motor skills performance on the Beery-VMI: A study of Canadian kindergarten children. Open Journal of Occupational Therapy,, 2014 2((2)): p. Article 4. 10.15453/2168-6408.1074

[b17] Durston,S. , van Belle,J. , & de Zeeuw,P. (2011).Differentiating frontostriatal and fronto-cerebellar circuits in attention-deficit/hyperactivity disorder. Biological Psychiatry,69(12),1178–1184. 10.1016/j.biopsych.2010.07.037 0006-3223 20965496

[b37] Epstein,J. N. , Erkanli,A. , Conners,C. K. , Klaric,J. , Costello,J. E. , & Angold,A. (2003).Relations between Continuous Performance Test performance measures and ADHD behaviors. Journal of Abnormal Child Psychology,31(5),543–554. 10.1023/A:1025405216339 0091-0627 14561061

[b52] Goldstand,S. , K.C. Koslowe , and S. Parush ,2005 Vision, Visual-Information Processing, and Academic Performance Among Seventh-Grade Schoolchildren: A More Significant Relationship Than We Thought?. Am J Occup Ther.,59(4),377–389. 16124204 10.5014/ajot.59.4.377

[b31] Goto,Y. , Hatakeyama,K. , Kitama,T. , Sato,Y. , Kanemura,H. , Aoyagi,K. , Sugita,K. , & Aihara,M. (2010).Saccade eye movements as a quantitative measure of frontostriatal network in children with ADHD. Brain & Development,32(5),347–355. 10.1016/j.braindev.2009.04.017 0387-7604 19505783

[b38] Jeannerod,M. , Arbib,M. A. , Rizzolatti,G. , & Sakata,H. (1995).Grasping objects: The cortical mechanisms of visuomotor transformation. Trends in Neurosciences,18,314–320. 10.1016/0166-2236(95)93921-J 0166-2236 7571012

[b27] Johnston,B. A. , Mwangi,B. , Matthews,K. , Coghill,D. , Konrad,K. , & Steele,J. D. (2014).Brainstem abnormalities in attention deficit hyperactivity disorder support high accuracy individual diagnostic classification. Human Brain Mapping,35(10),5179–5189. 10.1002/hbm.22542 1065-9471 24819333 PMC6869620

[b39] Johnstone,J. A. , & Ramon,M. (2011).Perceptual-motor activities for children: An evidence-based guide to building physical and cognitive skills.Human Kinetics.

[b48] Kandel,E. R. ,. . . (2000).Principles of neural science (Vol. 4).McGraw-hill New York.

[b5] Karcher,M. J. Connectedness and school violence: A framework for developmental interventions. Handbook of school violence, 2004: p. 7-42.

[b26] Krain,A. L. , & Castellanos,F. X. (2006).Brain development and ADHD. Clinical Psychology Review,26(4),433–444. 10.1016/j.cpr.2006.01.005 0272-7358 16480802

[b23] Leigh,R. J. , & Zee,D. S. (2015).The neurology of eye movements (Vol. 90).Oxford University Press 10.1093/med/9780199969289.001.0001

[b20] Lijffijt,M. , Kenemans,J. L. , Verbaten,M. N. , & van Engeland,H. (2005).A meta-analytic review of stopping performance in attention-deficit/hyperactivity disorder: Deficient inhibitory motor control? Journal of Abnormal Psychology,114(2),216–222. 10.1037/0021-843X.114.2.216 0021-843X 15869352

[b43] Lijffijt,M. , et al., A meta-analytic review of stopping performance in attention-deficit/hyperactivity disorder: deficient inhibitory motor control? 2005. 114(2): p. 216. 10.1037/0021-843X.114.2.216 15869352

[b15] Mahone,E. M. (2011).ADHD: Volumetry, motor, and oculomotor functions In Behavioral Neuroscience of Attention Deficit Hyperactivity Disorder and Its Treatment (pp. 17–47).Springer 10.1007/7854_2011_146 21732239

[b50] Martel,M. , Nikolas,M. , & Nigg,J. T. (2007).Executive function in adolescents with ADHD. Journal of the American Academy of Child and Adolescent Psychiatry,46(11),1437–1444. 10.1097/chi.0b013e31814cf953 0890-8567 18049293

[b54] Moore,T. and M.J.J.o.n. Fallah ,2004 Microstimulation of the frontal eye field and its effects on covert spatial attention., 91(1),152–162. 10.1152/jn.00741.2002 13679398

[b13] Mostofsky,S. H. , Lasker,A. G. , Cutting,L. E. , Denckla,M. B. , & Zee,D. S. (2001).Oculomotor abnormalities in attention deficit hyperactivity disorder: A preliminary study. Neurology,57(3),423–430. 10.1212/WNL.57.3.423 0028-3878 11502907

[b18] Mostofsky,S. , Lasker,A. G. , Cutting,L. E. , Denckla,M. B. , & Zee,D. S. (2002).Oculomotor abnormalities in attention deficit hyperactivity disorder. A preliminary study. 1. American Journal of Ophthalmology,133(1),176 10.1016/S0002-9394(01)01348-4 0002-9394 11502907

[b7] Munoz,D. P. , Armstrong,I. T. , Hampton,K. A. , & Moore,K. D. (2003).Altered control of visual fixation and saccadic eye movements in attention-deficit hyperactivity disorder. Journal of Neurophysiology,90(1),503–514. 10.1152/jn.00192.2003 0022-3077 12672781

[b30] Munoz,D. P. , & Everling,S. (2004).Look away: The anti-saccade task and the voluntary control of eye movement. Nature Reviews. Neuroscience,5(3),218–228. 10.1038/nrn1345 1471-003X 14976521

[b51] Nee,D. E. , Jahn,A. , & Brown,J. W. (2014).Prefrontal cortex organization: Dissociating effects of temporal abstraction, relational abstraction, and integration with FMRI. Cerebral Cortex (New York, N.Y.),24(9),2377–2387. 10.1093/cercor/bht091 1047-3211 23563962 PMC4184367

[b32] Nigg,J. T. (2001).Is ADHD a disinhibitory disorder? Psychological Bulletin,127(5),571–598. 10.1037/0033-2909.127.5.571 0033-2909 11548968

[b29] Overton,P. G. (2008).Collicular dysfunction in attention deficit hyperactivity disorder. Medical Hypotheses,70(6),1121–1127. 10.1016/j.mehy.2007.11.016 0306-9877 18215471

[b33] Pishyareh,E. , et al., (2012). Attentional bias towards emotional scenes in boys with attention deficit hyperactivity disorder. Iranian journal of psychiatry,7(2),93–93 22952552 PMC3428644

[b9] Pishyareh,E. , Tehrani-Doost,M. , Mahmoodi-Gharaie,J. , Khorrami,A. , & Rahmdar,S. R. (2015).A comparative study of sustained attentional bias on emotional processing in ADHD children to pictures with eye-tracking. Iranian Journal of Child Neurology,9(1),64–70. 1735-4668 25767541 PMC4322501

[b2] Polanczyk,G. V. , Willcutt,E. G. , Salum,G. A. , Kieling,C. , & Rohde,L. A. (2014).ADHD prevalence estimates across three decades: An updated systematic review and meta-regression analysis. International Journal of Epidemiology,43(2),434– 442. 10.1093/ije/dyt261 0300-5771 24464188 PMC4817588

[b6] Rapport,M. D. , Bolden,J. , Kofler,M. J. , Sarver,D. E. , Raiker,J. S. , & Alderson,R. M. (2009).Hyperactivity in boys with attention-deficit/hyperactivity disorder (ADHD): A ubiquitous core symptom or manifestation of working memory deficits? Journal of Abnormal Child Psychology,37(4),521– 534. 10.1007/s10802-008-9287-8 0091-0627 19083090

[b53] Rayner,K. ,2009 The 35th Sir Frederick Bartlett Lecture: Eye movements and attention in reading, scene perception, and visual search. ,62(8),1457–1506. 10.1080/17470210902816461 19449261

[b11] Ross,R. G. , Hommer,D. , Breiger,D. , Varley,C. , & Radant,A. (1994).Eye movement task related to frontal lobe functioning in children with attention deficit disorder. Journal of the American Academy of Child and Adolescent Psychiatry,33(6),869–874. 10.1097/00004583-199407000-00013 0890-8567 8083144

[b19] Rubia,K. , Overmeyer,S. , Taylor,E. , Brammer,M. , Williams,S. C. , Simmons,A. , & Bullmore,E. T. (1999).Hypofrontality in attention deficit hyperactivity disorder during higher-order motor control: A study with functional MRI. The American Journal of Psychiatry,156(6),891–896. 10.1176/ajp.156.6.891 0002-953X 10360128

[b46] Rubia,K. , et al.,Abnormal brain activation during inhibition and error detection in medication-naive adolescents with ADHD. 2005 162(6): p. 1067-1075. 10.1176/appi.ajp.162.6.1067 15930054

[b3] Satterfield,J. H. , Schell,A. M. , Nicholas,T. W. , Satterfield,B. T. , & Freese,T. E. (1990).Ontogeny of selective attention effects on event-related potentials in attention-deficit hyperactivity disorder and normal boys. Biological Psychiatry,28(10),879– 903. 10.1016/0006-3223(90)90569-N 0006-3223 2268691

[b8] Shiels,K. , & Hawk,L. W.,Jr. (2010).Self-regulation in ADHD: The role of error processing. Clinical Psychology Review,30(8),951–961. 10.1016/j.cpr.2010.06.010 0272-7358 20659781 PMC2952677

[b40] Sinclair,M. (2004).Pediatric massage therapy.Lippincott Williams & Wilkins.

[b16] Stray,L. L. , Stray,T. , Iversen,S. , Ruud,A. , Ellertsen,B. , & Tønnessen,F. E. (2009).The Motor Function Neurological Assessment (MFNU) as an indicator of motor function problems in boys with ADHD. Behavioral and Brain Functions,5(1),22. 10.1186/1744-9081-5-22 1744-9081 19450246 PMC2697164

[b47] Stuphorn,V.J.C.B. ,Neuroeconomics: cardinal utility in the orbitofrontal cortex? 2006 16(15): p. R591-R593. 10.1016/j.cub.2006.07.005 16890516

[b45] Swainson,R. , et al.,Cognitive control mechanisms revealed by ERP and fMRI: evidence from repeated task-switching. 2003 15(6): p. 785-799. 10.1162/089892903322370717 14511532

[b56] Treue,S.J.C.o.i.n. ,2003 Visual attention: the where, what, how and why of saliency. ,13(4),428–432. 10.1016/S0959-4388(03)00105-3 12965289

[b49] Wijetunge,G. ,. . . (2015).Prevalence of comorbidities in children with attention deficit and hyperactivity disorder at Lady Ridgeway Hospital for Children, Sri Lanka. Sri Lanka Journal of Child Health,44(2),77 10.4038/sljch.v44i2.7988 1391-5452

[b1] Wolraich,M. , Brown,L. , Brown,R. T. , DuPaul,G. , Earls,M. , Feldman,H. M. , Ganiats,T. G. , Kaplanek,B. , Meyer,B. , Perrin,J. , Pierce,K. , Reiff,M. , Stein,M. T. , Visser,S. , & the Subcommittee on Attention-Deficit/Hyperactivity Disorder, & the Steering Committee on Quality Improvement and Management (2011).ADHD: Clinical practice guideline for the diagnosis, evaluation, and treatment of attention-deficit/hyperactivity disorder in children and adolescents. Pediatrics,128(5),1007– 1022. 10.1542/peds.2011-2654 0031-4005 22003063 PMC4500647

[b22] Zee,D. S. , Leigh,R. J. , & Mathieu-Millaire,F. (1980).Cerebellar control of ocular gaze stability. Annals of Neurology,7(1),37–40. 10.1002/ana.410070108 0364-5134 6244772

